# Preparing Europe for Recurrent Autochthonous Dengue Transmission: Public Health Implications and Preparedness Priorities

**DOI:** 10.1016/j.lanepe.2026.101758

**Published:** 2026-06-24

**Authors:** Giuseppe Ippolito, Alimuddin Zumla

**Affiliations:** a Unicamillus International Medical University, Rome, Italy; b Institute of Infection, Immunity and Transplantation, University College London, and University College London Hospitals NHS Foundation Trust, London, United Kingdom

Dengue has historically been viewed in Europe primarily as a travel-associated infection. However, recurrent autochthonous outbreaks reported in several European countries indicate that dengue is now a recurrent vector-borne public health challenge requiring sustained preparedness and surveillance. Recent reports of outbreaks in Italy, Spain, and France indicate an evolving epidemiological situation characterised by repeated seasonal autochthonous transmission events in areas where competent Aedes spp vectors are established.^1^, ^2^, ^3^, ^4^, ^5^ Although public health specialists, arbovirus surveillance teams, and ECDC are well aware of these trends, awareness remains variable, clinicians, trainees, healthcare workers, travel medicine practitioners, policy makers, travellers, local communities, and the wider public, many of whom may still associate dengue predominantly with travel to tropical regions.

Although public health specialists and European Centre for Disease Prevention and Control (ECDC) keep abreast of all arboviruses, awareness remains variable among the local communities, healthcare workers including clinicians, medical students, junior and senior doctors, travellers, policy makers, and the wider public, many of whom may still associate dengue predominantly with travel to tropical regions.

Globally, dengue is the most rapidly expanding arboviral infection, with the World Health Organization documenting unprecedented dengue activity in recent years. Europe is increasingly exposed to several interacting factors driving dengue expansion globally, including climate change, vector establishment, urbanisation, population mobility, and global trade (Panel 1)^1^, ^2^, ^3^, ^4^, ^5^ Climate change is expanding and prolonging periods of climatic suitability for dengue transmission in parts of Europe that were already partially receptive to Aedes-borne transmission. However, temperature effects are non-linear: moderate warming may increase vectorial capacity, whereas extreme heat may reduce mosquito survival, fecundity, and fitness.^4^^,^^6^

Panel 1 **Conceptual framework for recurrent autochthonous dengue transmission in Europe: drivers, transmission processes, and public health implications.**

**Table:** 

Domain	Key component	Mechanisms	Public health implications
Global drivers and introduction pressures	Climate change	Rising temperatures, longer warm seasons, and changing rainfall patterns can increase periods suitable for vector survival and dengue transmission, although extreme heat may adversely affect mosquito fitness.	Increasing seasonal receptivity to dengue transmission in parts of Europe.
	Global travel and mobility	International travel results in repeated introduction of viraemic individuals into areas where competent vectors are established.	Importation pressure remains a prerequisite for most European outbreaks.
	Global trade and transport	International movement of goods has contributed to the introduction and spread of *Aedes albopictus* across Europe.	Expanding geographical opportunities for local transmission.
	Urbanisation and population density	Dense urban and peri-urban populations increase opportunities for human–mosquito contact and facilitate local amplification.	Increased outbreak potential in urban settings.
Ecological receptivity	Establishment of *Aedes albopictus*	*Aedes albopictus* is widely established across southern and central Europe and continues to expand into climatically suitable regions.	Larger populations are exposed to seasonal transmission risk.
	Seasonal vector abundance	Mosquito abundance increases during warmer months, creating seasonal windows of transmission opportunity.	Outbreak risk is strongly seasonal.
	Urban and peri-domestic breeding habitats	Artificial water containers and peri-domestic environments facilitate vector persistence and proliferation.	Source reduction remains central to prevention strategies.
Transmission dynamics	Introduction of dengue virus	Viraemic travellers introduce dengue virus into receptive ecological settings.	Early case recognition is essential.
	Local mosquito infection	Mosquitoes acquire infection following blood meals from viraemic individuals.	Vector surveillance complements clinical surveillance.
	Human–mosquito–human amplification	Repeated vector–host interactions can generate local transmission clusters.	Rapid intervention can limit outbreak growth.
	Delayed detection	Non-specific clinical presentations and unfamiliarity with dengue may delay diagnosis and response.	Improved clinician awareness is required.
Evolution of outbreak risk	Increasing outbreak frequency	Repeated introductions combined with ecological suitability may result in recurrent seasonal outbreaks.	Sustained preparedness is required even in years with low case numbers.
	Geographic expansion	Areas suitable for seasonal transmission may extend to additional regions and higher latitudes.	Surveillance systems may need geographic expansion.
	Multiple serotype introductions	Different DENV serotypes may be introduced over successive years.	Serotype surveillance remains important to assess future risks associated with secondary infections.
Public health preparedness	Integrated surveillance	Clinical, laboratory, entomological, environmental, and meteorological surveillance systems should operate in a coordinated manner.	Earlier detection and response.
	Diagnostics and clinical recognition	Timely laboratory confirmation and clinician awareness are critical.	Reduced delays in outbreak detection.
	Vector control	Source reduction, larval control, adult mosquito control, and innovative approaches such as Wolbachia may reduce transmission risk.	Core component of outbreak prevention and control.
	Risk communication and community engagement	Public participation is essential for sustainable vector control.	Improved community resilience and preparedness.
	Vaccination strategies	Future targeted vaccination strategies may be considered for selected high-risk populations and settings.	Potential adjunct to other prevention measures.

Conceptual framework illustrating the interacting global, ecological, epidemiological, and public health determinants contributing to recurrent autochthonous dengue transmission in Europe. The framework emphasises the sequence from viral introduction and ecological receptivity through local transmission and outbreak amplification, while highlighting opportunities for surveillance, prevention, and response.

The widespread establishment of *Aedes albopictus* across southern and central Europe is central to this changing epidemiological landscape.^2^^,^^4^^,^^5^ Global trade and transport networks have facilitated the introduction and spread of *Ae albopictus* across Europe, creating the ecological foundation upon which climatic suitability and travel-associated viral introductions now act. ECDC surveillance data demonstrate extensive distribution of *Ae albopictus* across Italy, France, Spain, the Balkans, and parts of central Europe.^2^ Increasing temperatures and prolonged warm seasons can enhance vector survival, biting frequency, and viral replication efficiency, thereby increasing the probability of seasonal transmission.^4^, ^5^, ^6^, ^7^ Recent analyses suggest that the interval between vector establishment and outbreak occurrence is shortening, indicating increasing ecological receptivity to arboviral transmission in Europe.^4^^,^^5^

Several recent outbreaks highlight this transition. France, Italy, and Spain reported confirmed autochthonous dengue transmission during 2023–2025, with reported annual case numbers varying substantially between countries and years^2^^,^^5^, ^6^, ^7^, ^8^, ^9^ (Table 1). The largest recent burden was reported in Italy in 2024, while 2025 activity was lower, emphasising that outbreak risk is shaped not only by climatic suitability and vector abundance but also by annual fluctuations in importation pressure, surveillance intensity, and public health response.^2^^,^^8^^,^^9^ Serological data from southern France suggest that limited under-recognition of dengue transmission may occur in some settings, although large undetected outbreaks remain unlikely under current surveillance conditions.^9^

**Table 1:** Autochthonous dengue transmission in Europe, 2023–2025: epidemiological characteristics, public health responses, and preparedness implications.

Year	Country/region	Reported autochthonous cases^a^	Main outbreak location(s)	Epidemiological characteristics	Public health response and lessons learnt	Implications for Europe
2023	France	45	Multiple departments including Alpes-Maritimes, Hérault, Pyrénées-Orientales, Gard and Drôme	Multiple geographically distinct outbreaks confirmed that local transmission can occur simultaneously across several regions during favourable seasons.	Enhanced surveillance, case investigation, vector control, and public communication were implemented.	Demonstrates recurrent seasonal transmission despite established surveillance and response systems.
2023	Italy	82	Lombardy, Lazio, Lodi, Anzio and surrounding areas	Largest burden reported in Europe during 2023, with evidence of efficient local transmission following imported index cases.	Integrated epidemiological, laboratory, and entomological investigations facilitated outbreak characterisation and response.	Confirms that sizeable outbreaks can occur in areas where *Aedes albopictus* is well established.
2023	Spain	3	Catalonia	Small local transmission cluster associated with imported infection.	Rapid detection and targeted vector-control measures limited further spread.	Illustrates ongoing vulnerability even where case numbers remain low.
2024	France	83	Multiple departments in southern France	Highest annual number of reported autochthonous cases to date in mainland France, involving multiple independent clusters.	Reinforced seasonal surveillance and rapid outbreak response activities.	Highlights increasing frequency of local transmission events and the need for sustained preparedness.
2024	Italy	213	Marche (Fano), Emilia-Romagna, Lombardy, Veneto, Tuscany and Abruzzo	Largest recent autochthonous dengue burden reported in mainland Europe, including a substantial outbreak centred in Fano.	Large-scale public health interventions included case finding, contact tracing, risk communication, and vector-control activities.	Demonstrates the potential for sizeable outbreaks when climatic suitability, vector presence, and viraemic importations coincide.
2024	Spain	8	Catalonia (Tarragona province)	Confirmed recurrent autochthonous transmission in a region previously affected in 2019 and 2023.	Prompt outbreak investigation and local control measures were implemented.	Reinforces the capacity for repeated seasonal transmission rather than geographic expansion into entirely new areas.
2025^b^	France	29	Multiple locations	Continued local transmission reported despite lower overall activity compared with 2024.	Ongoing surveillance and rapid public health response maintained.	Demonstrates substantial year-to-year variability in outbreak burden.
2025^b^	Italy	4	Localised clusters	Limited reported transmission compared with previous years.	Surveillance-driven detection enabled early intervention.	Illustrates the importance of importation pressure and seasonal ecological conditions in determining outbreak size.
2025^b^	Portugal (Madeira)	2	Madeira	Locally acquired dengue reported in a region with previous dengue activity.	Continued vector surveillance and public health monitoring.	Highlights the heterogeneity of dengue risk across European and EU-associated territories.

Summary of confirmed reported autochthonous dengue virus transmission events in Europe during 2023–2025 based on ECDC surveillance reports and published outbreak investigations. The table emphasises epidemiological characteristics, public health responses, and preparedness implications rather than providing a simple surveillance summary. Reported case numbers represent confirmed cases and should not be interpreted as estimates of total infections.

ECDC = European Centre for Disease Prevention and Control.

a Reported confirmed cases from ECDC surveillance reports and published outbreak investigations.

b Data available through ECDC seasonal surveillance updates.

As continental Europe enters the warmer months of 2026, public health authorities should remain vigilant for dengue transmission, recognising that outbreak risk is shaped by seasonal environmental suitability, vector abundance, and fluctuations in importation pressure. Dengue outbreaks place pressure on health-care systems with limited routine experience in dengue recognition and management. Reactive, localised containment alone is insufficient. Thus, Europe requires coordinated, climate-informed, and cross-sectoral preparedness for Aedes spp-borne diseases.^4^

Several gaps hinder proactive mitigation:

First, local vector-density and environmental thresholds associated with transition from self-limiting clusters to larger outbreaks remain insufficiently defined.
Second, the contribution of asymptomatic and mildly symptomatic infections to local transmission dynamics in European settings remains incompletely characterised.
Third, as multiple dengue virus serotypes are introduced into Europe over successive years, the long-term implications of repeated exposure and secondary heterologous infections warrant surveillance, although the current population-level risk for residents without travel-related exposure remains low.
Fourth, early clinical presentations are frequently non-specific, increasing the risk of delayed diagnosis and local amplification before vector-control interventions are implemented.
Fifth, fragmented integration between clinical, laboratory, entomological, environmental, and meteorological surveillance systems may hinder timely outbreak detection and response.
Finally, concurrent circulation of dengue, chikungunya, and West Nile virus supports the need for integrated arboviral surveillance and preparedness, while also creating diagnostic challenges in some settings. PCR-based confirmation remains central for acute case diagnosis.

Strengthening integrated surveillance systems, improving laboratory diagnostic capacity, harmonising vector surveillance, and enhancing clinician awareness should become immediate public health priorities. Climate-informed forecasting systems integrating meteorological, entomological, and epidemiological data may improve anticipatory preparedness and enable earlier intervention. Modelling studies from recent Italian outbreaks suggest that environmental extremes and delays in intervention implementation may substantially influence outbreak magnitude and duration.^10^

Novel interventions may complement, but are unlikely to replace, core public health preparedness measures. Wolbachia-based vector-control approaches have shown promising suppression of dengue transmission in endemic settings, although their utility in temperate European environments requires further evaluation.^11^ Similarly, newer dengue vaccines may have selective roles in high-risk populations or outbreak settings. However, their applicability in low-transmission European contexts remains uncertain.^12^

The repeated emergence, re-introduction, and local transmission of dengue in Europe should be viewed as an early warning signal of broader climate-sensitive infectious disease threats. Europe can no longer regard dengue only as an imported tropical infection. Coordinated European preparedness strategies, strengthened collaboration with dengue-endemic regions globally, increasing awareness among clinicians and travellers, and integration of climate science into infectious disease planning are essential to mitigate the risk of recurrent outbreaks and potential future expansion of dengue transmission in temperate regions (Supplementary Appendix Panel S1).

## Declaration of interests

Authors have a specialist interest in arboviral infections and One Health. They declare no conflicts of interest.
