# Supplementary material for: Preparing Europe for Recurrent Autochthonous Dengue Transmission: Public Health Implications and Preparedness Priorities
Source: Lancet Reg Health Eur. 2026 Jun 24;66:101758. doi: 10.1016/j.lanepe.2026.101758 (PMC13320452; doi:10.1016/j.lanepe.2026.101758)
Supplement: Supplemental Appendix [file mmc1.docx]

**Supplemental Appendix**

**Panel. Practical preparedness priorities for recurrent autochthonous dengue transmission in Europe**

| **Why dengue matters in Europe**  Autochthonous dengue transmission is now reported recurrently in several European countries. *Aedes albopictus* is established across large areas of southern and central Europe. International travel and global mobility continue to introduce viraemic individuals into receptive regions. Climatic suitability for seasonal transmission is increasing in many parts of Europe, although risk varies substantially between years.  **What clinicians, healthcare workers and communities should know**  Dengue should be considered in patients presenting with acute febrile illness during mosquito season, even without recent international travel. Early symptoms are often non-specific and may resemble influenza, COVID-19, chikungunya, West Nile virus infection, or other viral illnesses. Prompt laboratory confirmation and notification facilitate timely public health interventions. Awareness among clinicians unfamiliar with dengue remains an important component of preparedness.  **What public health authorities are prioritising**  Clinical, laboratory, entomological, environmental, and meteorological surveillance systems should be better integrated. Rapid case detection, outbreak investigation, and vector-control responses remain essential. Climate-informed early-warning systems, cross-border collaboration, harmonised surveillance approaches, and monitoring of DENV serotypes should be strengthened across Europe.  **What communities and travellers should know**  Elimination of mosquito breeding sites remains central to prevention. Personal protective measures, including repellents, protective clothing, and window screens, reduce exposure risk. Travellers returning from dengue-endemic regions may contribute to local transmission if viraemic while competent mosquito vectors are active. Public awareness campaigns can improve early recognition and healthcare seeking.  **Key knowledge gaps**  Important uncertainties remain regarding environmental thresholds for larger outbreaks, the contribution of asymptomatic and mildly symptomatic infections to transmission, the implications of repeated introductions of different DENV serotypes, the effectiveness of novel vector-control strategies in temperate European settings, and the interactions between dengue, chikungunya, West Nile virus, climate change, urbanisation, and human mobility. |
| --- |

**Legend: This supplementary Panel summarises practical preparedness priorities for recurrent autochthonous dengue transmission in Europe. It highlights implications for clinicians, public health authorities, communities, and travellers, while identifying key scientific uncertainties requiring further investigation.**
